# Label-free quantification of topical drug permeation in skin using sparse spectral sampling stimulated Raman scattering imaging

**DOI:** 10.1117/1.BIOS.3.4.043103

**Published:** 2026-08-31

**Authors:** Dandan Tu, Jackson Riseman, Yuxiao Wei, Priyanka Ghosh, Sam G. Raney, Markham C. Luke, Conor L. Evans

**Affiliations:** a Wellman Center for Photomedicine, Massachusetts General Hospital, Harvard Medical School, Boston, Massachusetts, United States; b Office of Research and Standards, Office of Generic Drugs, Center for Drug Evaluation and Research, U.S. Food and Drug Administration, Silver Spring, Maryland, United States

**Keywords:** coherent Raman imaging, skin, chemometric, hyperspectral imaging, spectral analysis, topical bioequivalence

## Abstract

**Significance:**

Stimulated Raman scattering (SRS) imaging has been used in evaluating topical drug product permeation. However, it has been mainly applied to a small group of active pharmaceutical ingredients (API) that have unique molecular bands which generate a prominent SRS signal against the background signal. The requirement of such unique molecular bands has been a large hurdle to generalizing SRS imaging to study a wide range of pharmaceutical molecules.

**Aim:**

To overcome this barrier, an imaging method based on sparse spectral sampling stimulated Raman scattering (S4RS) microscopy and multivariate analysis was developed in this study.

**Approach:**

Rather than relying on a single unique and strong peak, the API was specifically resolved using combined information from multiple wavenumbers across its spectrum. The workflow of applying this method to study APIs is demonstrated using metronidazole as an example. The sparse vibrational bands were first selected using Elastic-Net. Next, the metronidazole signal was unmixed from the compound signal of the skin-drug product mixture using multivariate curve resolution-alternating least squares (MCR-ALS).

**Results:**

The developed method demonstrated good capability in identifying the metronidazole signal in standard metronidazole-containing samples, including polymer films and skin samples treated with and without the drug product. This method enabled evaluation of the cutaneous pharmacokinetics (cPK) of metronidazole in *ex vivo* skin samples. Bioequivalence analysis was also completed for commercial drug products and laboratory-made control formulations containing metronidazole.

**Conclusions:**

The developed method enables the study of APIs that lack a unique prominent peak, thereby bridging SRS imaging to a broader range of drug products.

Statement of Translation This work developed an imaging method based on sparse spectral sampling stimulated Raman scattering (S4RS) microscopy and multivariate analysis. It enables tracking of active pharmaceutical ingredients (APIs), even those that lack a prominent, unique peak, in complex matrices (e.g., skin and formulation backgrounds). This method could be a useful tool for cutaneous pharmacokinetics (cPK) studies, helping to characterize the rate and extent of API permeation across the skin in a local area.

## 1 Introduction

Stimulated Raman scattering (SRS) imaging is a vibrational chemical imaging method that uses two synchronized lasers to probe a molecule’s vibrational signatures. Its intensity scales linearly with the number of molecules in the imaging volume.^1^ As SRS probes a molecule's intrinsic chemical structures, it allows for label-free molecular imaging. Conventional vibrational chemical imaging techniques, such as near-infrared (NIR) imaging, mid-infrared (MIR) imaging, and spontaneous Raman imaging have been widely used in detecting bulk and homogeneous samples. Efforts have been made to utilize these tools in imaging heterogeneous samples; however, limitations have been noted for these techniques. The spatial resolution of IR imaging is in the micrometer range, which is insufficient to identify and resolve signals from chemicals at a subcellular level. Confocal Raman imaging, which is often incorporated with various spatial sampling strategies, including point mapping, line mapping, and global imaging, has unavoidable tradeoffs among spatial resolution, acquisition time, and spectral resolution.^2^ In contrast to these conventional techniques, SRS offers subcellular spatial resolution, as well as high temporal resolution that enables video-rate imaging. Moreover, SRS is insensitive to samples’ autofluorescence, which is one challenge when using spontaneous Raman imaging for samples with high autofluorescence (e.g., skin).

SRS has been used in various pharmaceutical applications, especially those that require spatial-temporal resolution. Studies have used SRS imaging to show active pharmaceutical ingredient (API) uptake and localization at the subcellular level in living cells to understand the mechanism of APIs, such as tyrosine-kinase inhibitors (imatinib and nilotinib),^3^ and antimycin-type depsipeptides.^4^ Another study used SRS imaging to visualize ferrostatins in live cells, helping to better understand mechanism and aiding the development of new ferrostatins.^5^ In addition to visualizing API in cells, SRS has also been used to characterize the quality attributes of solid oral drug products. For example, SRS images are used to visualize the distribution of API and excipients in oral tablets, which helps to evaluate quality metrics such as particle sizes and distribution uniformity.^6^^,^^7^ Studying the permeation of topical drug products is another field that leverages the SRS imaging modality. There have been studies focusing on different aspects of topical drug product development and assessment, including studying the effect of excipients, the bioavailability (BA) of APIs, the permeation pathway of different formulations, and the bioequivalence (BE) of drug products. For example, SRS imaging helped investigate the permeation pathway of an antifungal API (terbinafine hydrochloride)^8^ and anti-inflammatory APIs (ketoprofen and ibuprofen)^9^ in skin. SRS has been used to show the BA of *trans*-retinol,^10^ ruxolitinib,^11^^–^^13^ 4-cyanophenol,^14^^,^^15^ lidocaine and loxoprofen,^16^ and zinc pyrithione and climbazole^17^ in different skin regions. Another study used SRS to investigate the kinetics of the uptake of different excipients (water, propylene glycol, and dimethyl sulfoxide) when they permeate human nails, which provides insight to develop topical drug products to better treat recalcitrant nail disease.^18^ More recently, SRS has also been used in a BE study of topical tazarotene creams, showing the capability of SRS to enable visualization of an API and semiquantitatively compare the API present in different skin microstructures.^19^

Current SRS-based pharmacokinetic studies have mainly been limited to a few APIs that either have unique chemical bands (e.g., alkyne, nitrile) that show a peak in the biological silent region or have a peak in fingerprint or high-wavenumber regions that is distinguishable from those of excipient or skin background. APIs meeting these conditions warrant that only the SRS signal specific to the API is measured. If neither of these conditions is met, another approach is to use deuterated molecules so that their C-D band or O-D band can provide a unique peak in the silent region. Availability of this unique and distinguishable peak^3^^–^^5^^,^^7^^–^^14^^,^^16^^–^^18^ has been the prerequisite of signal specificity where the unique peak enables univariate analysis that correlates intensity at a single wavenumber to the API. However, the presence of a unique vibrational band is rare across the various APIs in drug formulations, and deuteration could alter molecular features due to the ‘labeling’ process.

Hyperspectral SRS with multivariate analysis overcomes the limitation of the unique-peak univariate method. Multivariate SRS methods use SRS spectral information to resolve the API’s signal from the mixture, thereby avoiding reliance on special molecular bands, and making it generalizable for a wide range of molecules. Moreover, it is reported that results from multivariate analysis outperform univariate analysis for the same sample, which was demonstrated with model samples such as polystyrene (PS) and poly(methyl methacrylate) (PMMA) bead mixtures, $\mathrm{DMSO}/D_{2}O$ mixtures, and HeLa cell samples.^20^

Vibrational wavenumbers in an SRS spectrum can be probed using various approaches, such as sequential tuning of laser wavelengths,^21^^,^^22^ spectral focusing,^23^ and pulse shaping.^24^ SRS systems based on spectral focusing have limited tuning range (a couple of hundred wavenumbers) with one hardware setting at a time, which hinders rapid and free measurement across fingerprint, silent, and high-wavenumber regions. Pulse shaping methods generally have a relatively wide spectral resolution (about $10\;{\mathrm{cm}}^{-1}$), which limits its ability to resolve closely spaced peaks.^24^ An alternative is sequential rapid tuning of laser wavelengths, which can be deployed using all-fiber optical parametric oscillator (FOPO) laser that helps to achieve a good balance among tuning speed, wavenumber range, and spectral resolution. Specifically, the SRS system has a wide tuning range (700 to $3200\;{\mathrm{cm}}^{-1}$), good spectral resolution ($\leq 4\;{\mathrm{cm}}^{-1}$), and a fast tuning speed (5 ms).^22^^,^^25^ Moreover, all fiber laser systems can be more compact and thus resistant to external perturbations. Because each spectrum is collected by scanning wavenumber by wavenumber, the data acquisition time is directly related to the number of vibrational wavenumbers measured. Multiwindow sparse spectral sampling SRS (S4RS), which uses a sparse set of wavenumbers across the full spectrum, is a useful strategy to ensure sufficient spectral information and a short data acquisition time.^6^^,^^25^ In this work, the SRS system is based on a FOPO laser assisted with a computer program, which allows automated multicolor SRS measurement without manual operation.^25^

Multivariate analysis helps to extract qualitative and quantitative information from SRS spectral data. For example, there are various qualitative methods that have been widely used in classifying or grouping the measured sample into a chemical substance, such as principal component analysis (PCA), K-means algorithm, partial least square regression-discriminant analysis (PLS-DA), linear discriminant analysis (LDA), and soft independent modeling by class analogy (SIMCA).^26^^,^^27^ In the BA or BE study of drug products, quantifying or semiquantifying the amount of API is critical, and therefore, quantitative methods are needed. Classical least squares regression (CLS) and multivariate curve resolution (MCR) provide quantitative information and can be used for resolving components from a mixture.^26^ Because CLS requires the knowledge of all pure components in the mixture and their spectra, it is not suitable for resolving signal from a topical drug product permeation study, which contains complex components from both skin tissue and drug product samples. In contrast to CLS, the MCR method is advantageous for its capability of resolving spectra without prior knowledge of all pure component spectra. In general, MCR decomposes a mixture spectrum into the product of a pure spectrum profile of individual components and a concentration (or abundance) profile of these components.^28^ It iteratively optimizes the spectra of individual components and their concentrations according to the given mixture spectra. Multivariate curve resolution-alternating least squares (MCR-ALS) is the most widely used method, which uses the alternating least squares algorithm in iterative optimization. There has been success for MCR-ALS in extracting quantitative results from continuous full spectral data measured by hyperspectral SRS based on spectral focusing^29^^–^^32^ and pulse shaping.^33^^–^^36^ Despite these mentioned successful deployments of MCR-ALS, its performance in resolving S4RS spectra, which contains only a sparse set of wavenumbers rather than a continuous full spectrum, is unclear and warrants further study.

In this work, we developed a workflow to achieve label-free imaging of API permeation in skin based on multivariate analysis and S4RS. Metronidazole, an API typically contained in topical products to treat symptoms of rosacea, was used as a model molecule to demonstrate the developed method. In contrast to other spectral methods that utilize the entire continuous spectrum, S4RS requires selecting a sparse set of wavenumbers of the whole spectrum. The Elastic-Net method was used to determine the minimum number of wavenumbers that were representative of metronidazole and sufficient to distinguish it from background molecules in the skin and excipients in the formulations. The performance of MCR-ALS in resolving metronidazole from the skin’s and excipients’ background was evaluated. The quantitative performance of the MCR-ALS-assisted S4RS method was assessed using standard solution samples. Finally, a BE study of drug products containing metronidazole was carried out. The study included data collection from four donors’ skin samples using an S4RS microscope system, extraction of metronidazole abundance, and BE analysis of three sets of drug products.

## 2 Experimental

### 2.1 Materials

Metronidazole, poly(n-propyl methacrylate) (PPMA), poly(methyl methacrylate) (PMMA), poly(D,L-lactide-*co*-glycolide) (PLGA), poly(vinyl alcohol) (PVA), poly(D,L-lactide) (PLA), propylene glycol (PG, or 1,2 propanediol), dimethyl sulfoxide (DMSO), and other reagents were purchased from Sigma-Aldrich (St. Louis, Missouri, USA). 60×15  mm Corning™ Petri dishes, 1× Dulbecco’s phosphate-buffered saline with calcium and magnesium, and cotton-tipped applicators were purchased from Fisher Scientific™ (Hampton, New Hampshire, USA). Metrogel^®^ 0.75% metronidazole gel (NDC No. 66,993-962-45, Prasco Laboratories, LLC) and generic 0.75% metronidazole gel (NDC No. 00713-0637-37, Cosette Pharmaceuticals, Inc) were purchased from WEP Clinical (Morrisville, North Carolina, USA). Zorbentz™ lint-free disposable wipes were purchased from Thermo Fisher Scientific (Massachusetts, USA). Kimwipes^®^ low-lint wipers were purchased from U-LINE (Pleasant Prairie, Wisconsin, USA). Microdissecting surgical scissors and forceps were purchased from Roboz Surgical Instrument Co. (Gaithersburg, Maryland, USA). Sx micro-well glass bottom plates with 14-mm micro-wells and #0 cover glass were purchased from Cellvis (formerly In Vitro Scientific, Mountainview, California, USA). Absorptive neutral density (ND) filters (optical density = 1.0), were purchased from Thor-labs (Newton, New Jersey, USA)

### 2.2 Imaging System

We employed the same imaging system as used in previous studies.^19^^,^^25^ The Refined Laser Systems (RLS) Picus Duo all-fiber, near-infrared light source with integrated optical parametric oscillator (OPO) and amplitude-modulated, tunable wavelength pump beam was employed. The pump and Stokes beams were coupled using external optics. The combined beam was aligned into an Olympus (now Evident) FV3000 inverted confocal laser scanning microscope. A 20X objective with a 0.8 NA was used. A SRS photodiode detector from Applied Physics and Electronics (APE) was positioned on top of the microscope condenser (0.55 NA) to detect the Stokes beam transmitted through samples. Long-pass optical filters were placed between the microscope condenser and the detector to block pump beam light and only detect the Stokes beam. The APE detector had an integrated lock-in amplification module to demodulate the measured stimulated Raman gain (SRG) signal. A Python program employing the Olympus remote development kit was used to communicate with the microscope’s Fluoview software and control the laser. This helped to achieve an automated spectral data collection. The spectral performance of the system was assessed using naproxen powder. The peak position of $1396\;{\mathrm{cm}}^{-1}$ was used as the reference. If wavenumber-drift from this value was observed in the naproxen powder, the measured SRS spectra would be shifted to compensate for the drift.

### 2.3 Metronidazole Standard Polymer Film Preparation

The metronidazole-embedded polymer film was formulated by dissolving metronidazole powder and PLA in DMSO, then drying in a convection oven until forming pieces of solid film. To produce the precursor solution, 30 mg of PLA polymer and 2.5 mg of metronidazole were mixed to achieve a drug to polymer concentration of 7.5% w/w. Then, 600 mL of DMSO solvent was added to make the PLA to DMSO concentration of 5% w/v. The solution was vortexed, sonicated for 20 min, vortexed again to ensure the reagents were fully dissolved, and then cast at a 160  μL volume into each 10-mm diameter well of poly(dimethyl siloxane) (PDMS) molds pressed onto large glass slides. The filled mold(s) were then placed in a tabletop convection oven set to 70°C for 2 h, or until films were completely dry. After drying, the films were placed in a fume hood to cool for 10 min; then, the PDMS molds were gently peeled off the glass slides using a razor blade to lift them off from the edges and using forceps to handle them. If the films were still soft and not completely cooled, an additional 10 to 20 min of cooling and hardening in a fume hood would be carried out before peeling them off. The peeled-off films were then turned upside down to rest on the glass slide by their mold-formed edges, and then, left in a fume hood for an additional hour. Further drying and cooling prevented the films from sticking to each other during storage. The same steps used to prepare the above 7.5% w/w film were used to prepare films with drug-to-polymer concentrations of 0%, 3.75%, 7.5%, and 10% w/w. The obtained films were then placed in a sealed container with desiccant pellets and stored for future use at 4°C.

### 2.4 Ex Vivo Human Skin Preparation

The use of de-identified human skin tissue was approved by the Massachusetts General Hospital institutional review board (IRB) as “exempt, nonhuman” research. The *ex vivo* human abdominal skin originated from elective abdominoplasty procedures via an approved protocol. The skin was sectioned into 1.5  cm×1  cm pieces using the method mentioned previously^19^^,^^37^ and stored at −20°C.

To prepare for imaging of standard skin samples, very thin slices (thickness <60  μm) of skin tissue were prepared to allow maximum light transmission and also to enable imaging at the skin’s edge. To prepare the sample, a 1.5  cm×1  cm piece skin (41-year-old female) was placed on top of dry ice to keep it frozen. After the tissue had completely hardened after freezing, a scalpel was used to shave a thin layer from the epidermis side of the tissue.

The following procedure was used to prepare skin samples for the BE study: four donors (one 41 year-old female, one 64 year-old female, one 38 year-old female, and one 52 year-old male) were used. The tissue trimming procedure was similar to that of our previous study.^19^^,^^37^ In brief, the preportioned frozen skin samples were thawed and then trimmed from the dermis side using surgical scissors and forceps until barely translucent under room light. When finished trimming, the skin was divided into three 0.5×1  cm pieces using a scalpel.

### 2.5 Standard Sample Imaging

The following procedure was used to collect the reference spectra of pure samples and skin samples, and all spectra were collected from 1000 to $1600\;{\mathrm{cm}}^{-1}$ with a step size of $4\;{\mathrm{cm}}^{-1}$. The above-prepared, nontreated, very thin slices of skin (thickness <60  μm) were used to collect spectra for the melanin- and nonmelanin-containing portions of skin. The thin layer of skin was placed in a glass-bottom six-well plate with the epidermis side contacting the glass, closest to the objective, for imaging of the nonmelanin region. The ROI was chosen to ensure that an empty “air” region appeared within the same field of view (FOV) as the skin. The nonmelanin skin spectrum was collected by subtracting the spectrum of the empty background region from the spectrum of a rectangular region of the untreated skin. Conversely, the thin layer of skin was placed in the plate with the subcutaneous side contacting the glass, in which high concentrations dark melanin was present in SRS images. The spectrum of rectangular regions of this melanin was collected. The final melanin spectra were obtained after subtracting the empty background. Metronidazole was dissolved in water to prepare a 7.5  mg/mL solution. The solution was sandwiched between a glass slide and a coverslip using spacer tape to maintain a constant sample thickness (around 120  μm). An ROI was selected to include an empty area and the solution area. The final metronidazole spectrum was obtained after subtracting the background spectrum of the empty area. The same process was used to collect the spectra of propylene glycol and sodium hydroxide (2 M sodium hydroxide in water). All other excipients (carbomer 940, edetate disodium, propyl 4-hydroxybenzoate, and methyl 4-hydroxybenzoate), which are molecules in the metronidazole formulations studied in this paper, were measured using dry powder. A trace amount of excipient powder was spread on a coverslip for SRS imaging. The regions in which the powder was in focus were selected to form the raw spectra. The regions where no powder appeared were selected to form the background spectrum. The final spectrum of the excipient was obtained by subtracting the background spectrum from the excipient raw spectrum.

To classify human skin with and without application of the metronidazole drug product, the above-prepared very thin slices of skin (thickness <60  μm) were used. One group of samples was left untreated, and the other group was treated with 1  μL of 0.75% metronidazole gel RLD (Metrogel^®^). The skin was placed in a glass-bottom six-well plate with the epidermis side contacting the glass. Each sample was imaged at its edge so that the FOV contained an empty area and the sample. The sample was imaged from 1000 to $1600\;{\mathrm{cm}}^{-1}$ with a step size of $4\;{\mathrm{cm}}^{-1}$. The “bare skin” spectrum was obtained by subtracting the background spectrum from the untreated skin spectrum. The “skin + drug product” spectrum was obtained by subtracting the background spectrum from the Metrogel^®^-treated skin spectrum.

In the study for quantitative analysis of metronidazole in standard samples, the following samples were imaged, with all samples imaged from 1000 to $1600\;{\mathrm{cm}}^{-1}$ with a step size of $4\;{\mathrm{cm}}^{-1}$: first, films with varying metronidazole concentrations (0%, 3.75%, 7.5%, and 10% w/w) were used; multiple films were positioned close to each other, and then, an ROI with two to three different films focused in one FOV was imaged. The collected hyperspectral image stacks were used to analyze the API gradient in the FOV. Second, the above-prepared, nontreated, very thin slices of skin were used to collect images to visualize the API distribution in different skin samples. Specifically, one piece of skin was treated with 1  μL propylene glycol, and the other was treated with 1  μL of 0.75% metronidazole gel RLD (Metrogel^®^). The pieces were positioned close to each other with an empty gap between them. The collected hyperspectral image stacks were processed to show the API distribution in these skin samples. Third, different concentrations of metronidazole in water were imaged. Each concentration solution was sandwiched between a glass slide and a coverslip using spacer tape (120  μm approximate thickness). The spectra of each solution, after subtracting the empty-area background spectra, were collected.

### 2.6 Bioequivalence Experiment

In the BE study, four groups of data were collected. Specifically, two separate groups of data, labeled as R1 and R2, were collected from skin treated with 0.75% metronidazole gel RLD (Metrogel^®^). Another group of data was collected from skin treated with the generic 0.75% metronidazole gel (Cosette Pharmaceuticals, Inc). Data for the last group were collected from skin treated with the laboratory-made formulation containing 0.75% metronidazole (solvent was 90:10 v/v water/propylene glycol).

The BE experiment procedures employed in this study are similar to those previously mentioned.^19^ More specifically, each drug permeation imaging experiment was organized in four wells of a six-well glass-bottom plate, with three wells containing skin samples and one well containing an ND filter. Two treatment groups were tested in one experiment, and two pieces of the three 0.5  cm×1  cm skin samples were used. Depending on the experiment to be performed in the randomized series, the metronidazole RLD, the generic product, or the laboratory-made formulation was applied to the epidermis side of the skin. For each piece of skin, a 5  μL volume of drug was dispensed. Then, each drug-applied piece of skin was placed epidermis side down into a well. A small section near the center of a 7.5% w/w metronidazole-embedded PLA polymer film was cut to use as an imaging standard. The piece of film was placed in the third well, and the third piece of skin, without the formulation, was placed epidermis-side down on top of the film. Each piece of skin was gently flattened using PBS-moistened cotton-tipped applicators.

Three imaging ROIs were chosen for each drug product-applied piece of skin. Each ROI was composed of an image stack starting at the focus of visible corneocytes (0  μm) to 64  μm deep in the skin, with a step size of 16  μm. One ROI was chosen for the polymer standard film skin, and this image stack (0 to 64  μm) was acquired starting at the focus of one step size away (closer to epidermis) from the polymer film’s brightest high-wavenumber signal (negative 16  μm from highest signal became 0  μm point). This approach helps prevent the optimal focal plane from shifting outside the imaging stack range, in cases where air was initially trapped between the polymer film and the well plate glass bottom and was gradually removed during the course of the experiment. One ROI was chosen for the ND filter with no skin, and this ROI was acquired at a single depth, focused on the plate’s glass. The ND filter was used to attenuate the transmitted light from the well with no skin to prevent saturation of the SRS detector. Signal acquired from this ND filter ROI was used to track the background SRS intensity, which is useful for monitoring optical system-induced signal fluctuations and for baselining spectra collected in other ROIs. All ROIs were registered at a scan size of 512×512  pixels, a pixel integration time of 10  μs, and with the FV3000 microscope’s Z-drift compensation (ZDC) mechanism activated. The protocol scanned all eight ROIs as one cycle, and switched wavenumber through the wavenumber sequence of 2870, 1660, 1592, 1500, 1496, 1468, 1460, 1452, 1396, 1392, 1388, 1376, 1372, 1296, 1288, 1284, 1192, 1124, and $1032\;{\mathrm{cm}}^{-1}$, going through the list seven times for a total of 133 cycles (19×7). The number of wavenumber list loops was chosen so that the experimental length would be ≤7  h. For the duration of each permeation experiment, the well plate was placed in a microscope stage-top incubator (Tokai Hit INUG2F-IX3D) at 90% humidity and a temperature of 32°C.

### 2.7 Multivariate Analysis

Multivariate analysis in this study was programmed in Python using Jupyter Notebook. Support vector machine (SVM) was used to classify the spectra from skin with and without application of the metronidazole drug product. Each spectra was obtained by subtracting the spectra of a blank area within the same field of view of the skin (background spectra) from the raw spectra of the skin. All spectral data were split, with 70% used as training data and 30% as test data. ElasticNet was used to determine a group of sparse wavenumbers representative of the metronidazole molecule. The spectra from the metronidazole solution (water as solvent) and the spectra from the nonmelanin skin region were used as the two components in ElasticNet analysis, with alpha=1 and L1 ratio=0.5. Sparse wavenumbers were determined using the training data, and then, the selected sparse wavenumbers were tested using the test data. The SVM and ElasticNet functions in scikit-learn were used.

Multivariate curve resolution-alternating least squares (MCR-ALS) was used to resolve and decompose metronidazole’s signal from the mixture’s signal. In general, MCR decomposes a mixture spectrum (M, with m×p dimensions) into the product of spectra of individual components (S, with m×n dimensions) and the concentration (or abundance) of these components (C, with p×n dimensions). Here, p equals the number of wavenumbers in one spectrum, m equals the number of mixture samples, and n equals the number of individual components used in the decomposition. T means the transpose of matrix S. As described in Eq. (1), the signal from the mixture can be expressed as $C\cdot S^{T}$ plus the residual matrix of error (E). In this study, spectra of the following components were used as the input reference spectra in MCR-ALS analysis: metronidazole, nonmelanin skin, melanin skin, PG, and blank background.

$$M_{m\times p}=C_{m\times n}\cdot {S^{T}}_{p\times n}+E_{m\times p},$$ (1)

$$S=[s_{\text{Metronidazole}},s_{\text{Non}-\text{melanin}-\text{skin}},s_{\text{Melanin}},s_{\mathrm{PG}},s_{\text{Background}}].$$ (2)

The pyMCR function from a previous work^38^ was used for MCR-ALS analysis. Constraints of non-negative concentration and non-negative pure component’s spectrum were applied. After inputting the mixture matrix M and the reference spectra of each component, MCR-ALS analysis outputs S and C matrices. S contains the fitted spectra of each component. C contains the abundance of each component in the mixture spectrum, which represents the relative contribution of the component’s spectrum to the intensity of the optimized mixture spectrum. Specifically for metronidazole, $c_{\text{Metronidazole}}$ can be used to reflect the quantity of metronidazole present in the area. In addition, a resolved metronidazole spectra ($s_{\text{Metronidazole}}^{\prime }$) can be acquired by multiplying the abundance $c_{\text{Metronidazole}}$ to the fitted spectra $s_{\text{Metronidazole}}$ as shown in Eq. (3). Resolving $s_{\text{Metronidazole}}^{\prime }$ helps to reduce the signal contributions attributed to other components present in the mixture and thus had an improved specificity to metronidazole.

$$\text{Resolved metronidazole contribution }s_{\text{Metronidazole}}^{\prime }=c_{\text{Metronidazole}}\cdot s_{\text{Metronidazole}}.$$ (3)

The hyperspectral image stack data were processed in the following manner: one image stack with 512×512  pixels has 262,144 spectra (m=262144) that need to be resolved; to process pixel by pixel, the 512×512 image with 17 wavenumbers (frames) were first folded to a matrix of 262144×17. After MCR-ALS analysis, the output $C_{262144\times 5}$ and $S_{17\times 5}$ were acquired. The metronidazole abundance $c_{\text{Metronidazole}}$ could be extracted, and the resolved metronidazole spectra $s_{\text{Metronidazole}}^{\prime }$ could be calculated. The peak intensity (e.g., $1192\;{\mathrm{cm}}^{-1}$) of the resolved metronidazole spectra could be extracted and formed a matrix with a dimension of 262144×1. The metronidazole abundance or peak intensity matrix was then refolded to 512×512, serving as an intensity map showing the resolved SRS signal of metronidazole and demonstrating metronidazole’s distribution.

### 2.8 Pharmacokinetic Analysis

The BE analysis compared AUC values and $C_{\max}$ values and Cmax values of different topical treatments. The following process was used to extract the AUC and $C_{\max}$ values from the collected data. The 133-cycle image volumes collected at the ROIs applied with formulations were used to generate intensity-time profiles. Each depth of the image volume was processed individually. One depth of each imaging volume (133 frames) was split into seven segments, with each segment containing 19 frames. The image frame collected at $2870\;{\mathrm{cm}}^{-1}$ was used to show the lipid-rich microstructure of the skin. The image frame collected at $1660\;{\mathrm{cm}}^{-1}$ exploited the signal of the amide bond, which was mainly attributed to and could help to show the abundance of skin protein. The remaining 17 image frames were folded and analyzed by MCR-ALS. The metronidazole abundance matrix was then refolded to a 512×512 image. This process was applied to all segments of the 133 frames, which generated seven images showing the metronidazole distribution at different time points after applying the formulation on skin. The pixels of each image were averaged to obtain an API intensity-time profile. The API intensity-time profile was then used to calculate the AUC value and $C_{\max}$ value, using noncompartmental analysis (NCA), which was completed in an automated fashion in R using the library NonCompart.^39^

AUC values and $C_{\max}$ values from all replicates of different donors and samples using the drug formulations were used in BE analysis. A similar method as described in our previous work^19^ was used for BE analysis. The confidence interval when comparing a pair of treatment groups was calculated, and two drug products were considered bioequivalent when the upper and lower limits were within the limits of 0.8 to 1.25.

## 3 Results and Discussion

### 3.1 Selection of Sparse Wavenumbers for Metronidazole

Metronidazole is a synthetic nitroimidazole derivative, which has a LogP value of −0.02, is soluble in water, and has antiprotozoal and antibacterial effects.^40^ Metrogel^®^ (metronidazole topical gel) 0.75% is a reference drug product that is usually used for the treatment of rosacea. BE studies using metronidazole-containing drug products have been performed using different methods, such as dermal microdialysis (dMD), IVPT, tape stripping combined with liquid chromatography-mass spectrometry, and confocal Raman microscopy.^40^^–^^42^ Notably, these methods have limited capability to show high-resolution spatiotemporal images of metronidazole distribution in the skin. One study used confocal Raman and SRS to visualize metronidazole in skin; however, only a single peak was used.^42^ In this work, the S4RS imaging method, combined with multivariate analysis, was used to generalize the SRS imaging method to a broad range of molecules.

In previous topical drug product studies using SRS imaging,^12^^,^^19^ the selected APIs generally have at least one primary peak, which is prominent and has high contrast over the skin’s and any non-API excipients’ backgrounds. The availability of such a primary peak ensured the specificity of the SRS signal to the API when imaging it in the complex skin-formulation matrix. Figure 2(a) shows the SRS spectra of metronidazole at the concentration present in the drug product. At the tested concentration of metronidazole dissolved in water without formation of any crystals, there is no such single primary peak that has high contrast over the skin’s background. Moreover, the metronidazole peaks also overlapped with the spectra of excipients that could be present in Metrogel^®^ [shown in Fig. 2(b)]. Therefore, an alternative method was needed for imaging metronidazole, an example of an API lacking unique primary peaks.

**Fig. 1:**
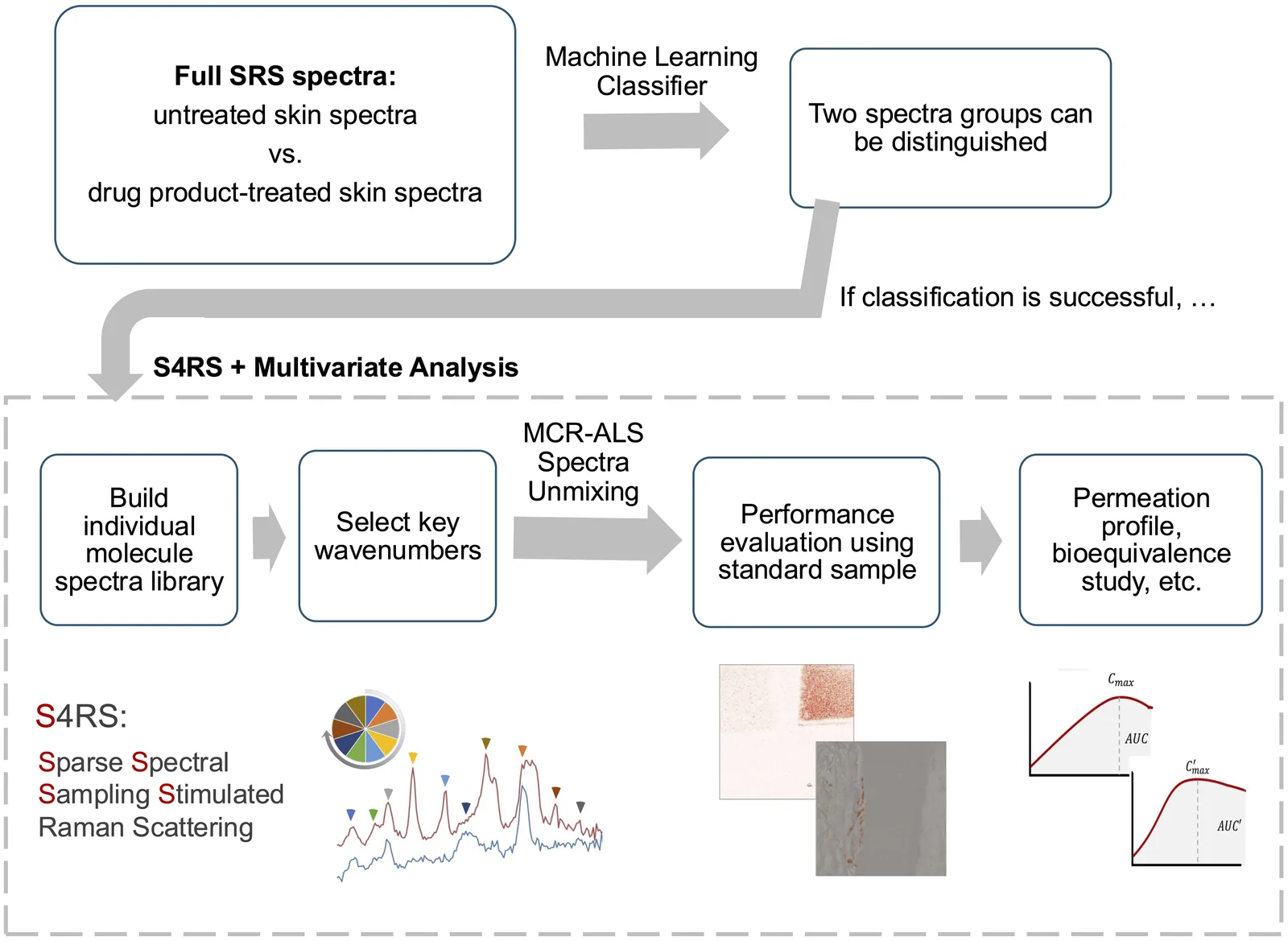
Overview of steps in developing an imaging method based on sparse spectral sampling stimulated Raman scattering (S4RS) microscopy and multivariate analysis.

**Fig. 2:**
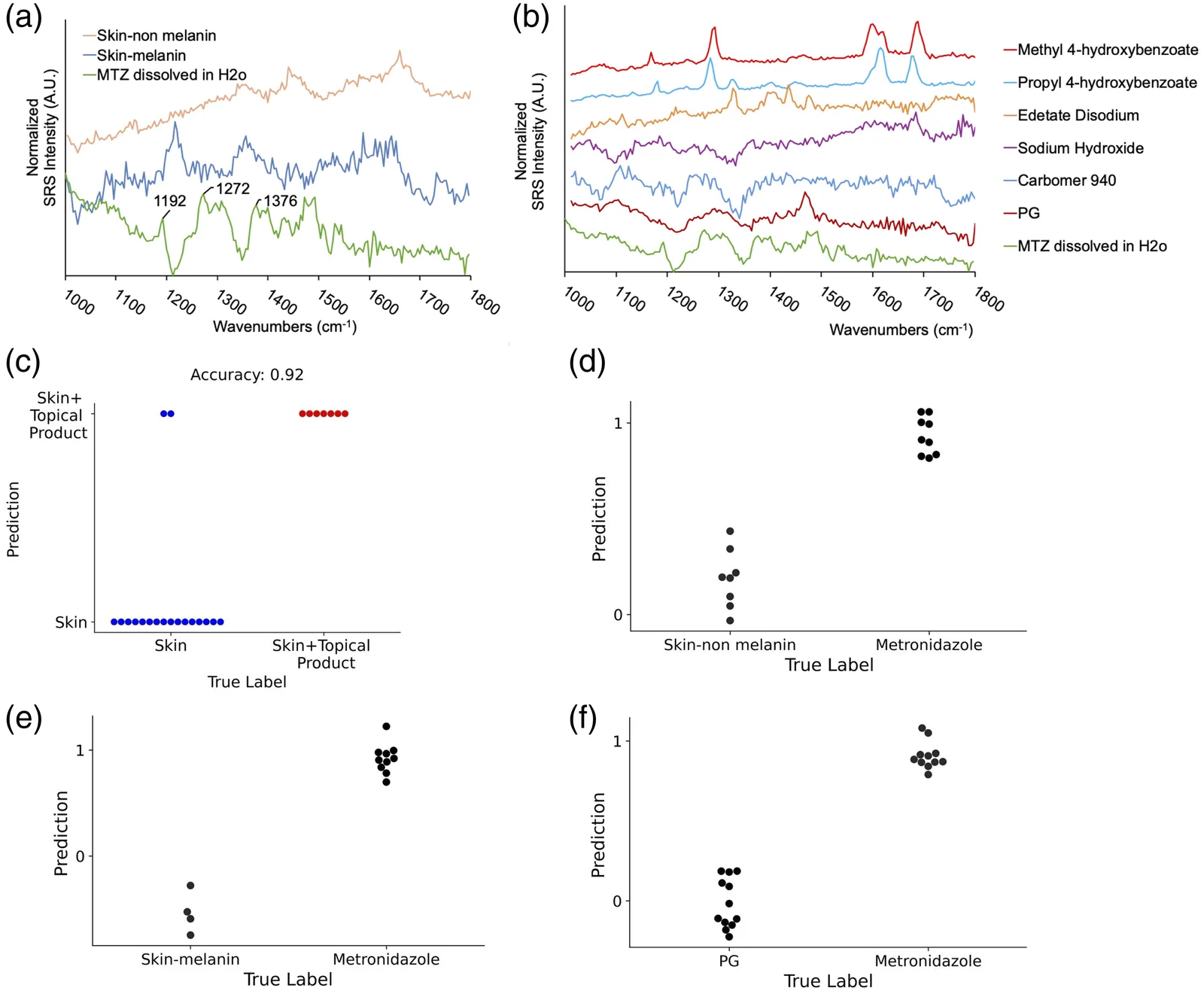
(a) SRS spectra of nonmelanin skin, skin melanin, and 0.75% metronidazole dissolved in water. (b) SRS spectra of excipients present in the 0.75% Metrogel^®^ drug product. (c) Classification of skin with and without the 0.75% Metrogel^®^ drug product using the SVM method. Full-spectrum data were used for the classification. The SVM model was trained using 70% of the data, and the plotted results used the remaining 30% of test data. (d)–(f) Elastic net results using the 17 selected peaks for differentiating metronidazole from nonmelanin skin, skin melanin, and propylene glycol (PG). The elastic net model was trained using 70% of the data, and the plotted results used the remaining 30% of test data. The predicted value of 0 indicates no presence of metronidazole, and the value of 1 indicates the presence of metronidazole.

A workflow was developed to utilize the S4RS-based method in the BE study of an API in drug products. The overview of general steps was shown in Fig. 1. The detailed workflow was shown in Fig. S1 in Supplementary Material. The workflow is composed of three sections. In the first section, a machine learning algorithm was used to evaluate whether using the full spectrum can distinguish skin applied with the drug product from bare skin. The full spectrum contains the maximum information that can be used, and additionally, machine learning is a powerful tool that can be trained to achieve high-accuracy classification and group differentiation. Specifically, SVM was used to classify bare skin and skin applied with the 0.75% Metrogel^®^ drug product. SVM is a machine learning algorithm that finds an optimal hyperplane that separates data into classes. It has been widely used in classification tasks and has shown good performance. If these two groups (bare skin and skin applied with the drug product) can not be classified by SVM, this likely indicates high similarity between the two spectra groups, and therefore, a significant challenge in identifying the API from the skin background. If this is the case, an alternative method should be used instead. If these two groups can be classified, it suggests that the SRS spectra may be used to identify molecules in the drug product against the skin background. Figure 2(c) shows that SVM successfully classified the two skin groups, allowing for the subsequent assessment of the feasibility of using spectral information to identify molecules in the drug product.

In the second section of the workflow, a subset of wavenumbers from the full spectra was selected to identify the API from the complex skin-formulation matrix. S4RS is a method that uses a sparse set of wavenumbers that contain sufficient spectral information for spectral separation.^6^^,^^25^ A crucial factor determining the performance of S4RS is the selection of the minimum number of required wavenumbers. A good selection of wavenumbers should ensure a short data acquisition time (fewer wavenumbers) while maintaining signal specificity (better capability to identify the API). Rather than relying on subjective selection through visual comparison of spectra or simple thresholding, a machine-learning-based feature selection algorithm was employed to identify the wavenumbers that distinguish an API from non-API components. Specifically, elastic net is used to select the sparse set of wavenumbers from the full spectrum. Elastic net is a regularized regression method that combines the strengths of both Lasso and Ridge regressions. During the regularized regression process, it keeps important features and removes irrelevant features by setting the coefficients of irrelevant features close to zero. Notably, the SRS spectral data used in this study has a larger number of wavenumbers (predictor number, p) than the number of collected pure component spectra (observation number, n). Elastic net is more suitable for these “large p and small n” data and has been widely used in feature selection in spectral data.^43^^–^^46^ When imaging skin applied with the 0.75% Metrogel^®^, the sample in the field of view may contain skin, metronidazole, and all excipients of the drug formulation. Among these components, skin is the most abundant, and it would potentially have the most significant effect in interfering with metronidazole measurement. Considering this, elastic net was first used to identify important wavenumbers for distinguishing metronidazole from the skin (nonmelanin region). Using the elastic net, a total of 17 wavenumbers were selected: 1592, 1500, 1496, 1468, 1460, 1452, 1396, 1392, 1388, 1376, 1372, 1296, 1288, 1284, 1192, 1124, and 1032. As shown in Fig. 2(d), the 17 wavenumbers successfully distinguished metronidazole and the skin (nonmelanin region). Follow-up elastic net regressions were carried out to confirm that these 17 peaks were also sufficient to distinguish metronidazole from the other components. Figures 2(e) and 2(f) shows that metronidazole could be differentiated from medium-abundance components, such as skin melanin and PG. In addition, metronidazole could be distinguished from low-abundance components, such as sodium hydroxide, carbomer 940, edetate disodium, propyl 4-hydroxybenzoate, and methyl 4-hydroxybenzoate (Fig. S2 in Supplementary Material). The above results confirmed that the selected wavenumbers were capable of differentiating metronidazole from all other non-API components. Notably, these selected 17 peaks included $1192\;{\mathrm{cm}}^{-1}$ (attributed to C─N stretching) and $1376\;{\mathrm{cm}}^{-1}$ (attributed to aromatic C─N stretching),^47^^,^^48^ which are representative strong peaks for dissolved metronidazole. This confirms that elastic net can identify a subset of wavenumbers representative of the API.

In the third section of the workflow (Fig. S1 in Supplementary Material), method validation was performed on standard materials and skin samples with known, controlled compositions. This part is important for optimizing and evaluating the multivariate analysis method and the selected wavenumbers. After confirming the performance of the multivariate analysis method, it was then applied to actual BA or BE experiments. Details about the third section of the workflow are discussed in the following sections.

### 3.2 Multivariate Analysis on Standard Samples

Measuring an API in a complex matrix, in which interfering components such as the skin background and excipients in the drug products are present, requires a method to resolve the portion of the signal specifically correlated with the API. MCR-ALS is a multivariate analysis method that has been widely used to decompose a mixture spectrum into pure components and shows the quantitative information of each component. MCR-ALS mainly uses full continuous spectral data to extract quantitative results. However, resolving components using MCR-ALS with a sparse number of wavenumbers (<30) has not been studied, and thus, MCR-ALS combined with S4RS was carefully evaluated in this work.

A step-by-step evaluation process was used to validate the MCR-ALS method in resolving the metronidazole signal from a mixture. First, the method was tested using standard API-embedded polymer samples with known, stable, and controllable compositions. Starting with these samples enabled the use of a predefined mixture composition for imaging, thereby enabling knowledge of expected outputs. After confirming the method with standard polymer samples, the next step was to test our analysis method using skin treated with the drug product. Compared with the standard polymer sample, skin with the drug product sample contains more variables. Because formulation permeates into skin, its composition, including the abundance of metronidazole, varies over time at a given imaging spot. Despite the time-variant compositions, skin with the drug product was an important standard sample for evaluating our analysis method, as it contained all molecules (the API and interfering components) present in the samples used in actual BA/BE studies.

The first standard sample used in this work was a transparent, thin polymer film. The polymer film had the advantages of being optically clear, being stable in composition when positioned on skin, containing simple components (API and polymer), and being controllable in component concentrations. Procedures to make a polymer standard film embedded with a lipophilic API have been reported in our previous work.^19^ However, because metronidazole is hydrophilic, the previous method failed to make an optically transparent and homogeneous film. A new method was developed to manufacture a polymer standard film suitable for hydrophilic molecules, such as metronidazole. The detailed process for developing the method is described in the Supplementary Material. Among the tested combinations of solvent and polymer base, dimethyl sulfoxide (DMSO) and PLA (poly(D,L-lactide) or poly-lactic acid) were the optimal combination because they produced a solid, inert, transparent, and clear metronidazole-embedded film. DMSO showed good ability to solvate metronidazole (shown in Table S1 in Supplementary Material). A high solubility of metronidazole was helpful to increase the upper concentration limit of metronidazole contained in the film, allowing testing of standard films covering a wider concentration range of metronidazole. The mixture of PLA and a high concentration of metronidazole was well dissolved in DMSO, and the resulting film didn’t undergo moisture-induced disintegration, unlike the film made with PVA as the polymer base. Therefore, the metronidazole-embedded film made with DMSO and PLA was used as the standard polymer film in the following experiment.

**Fig. 3:**
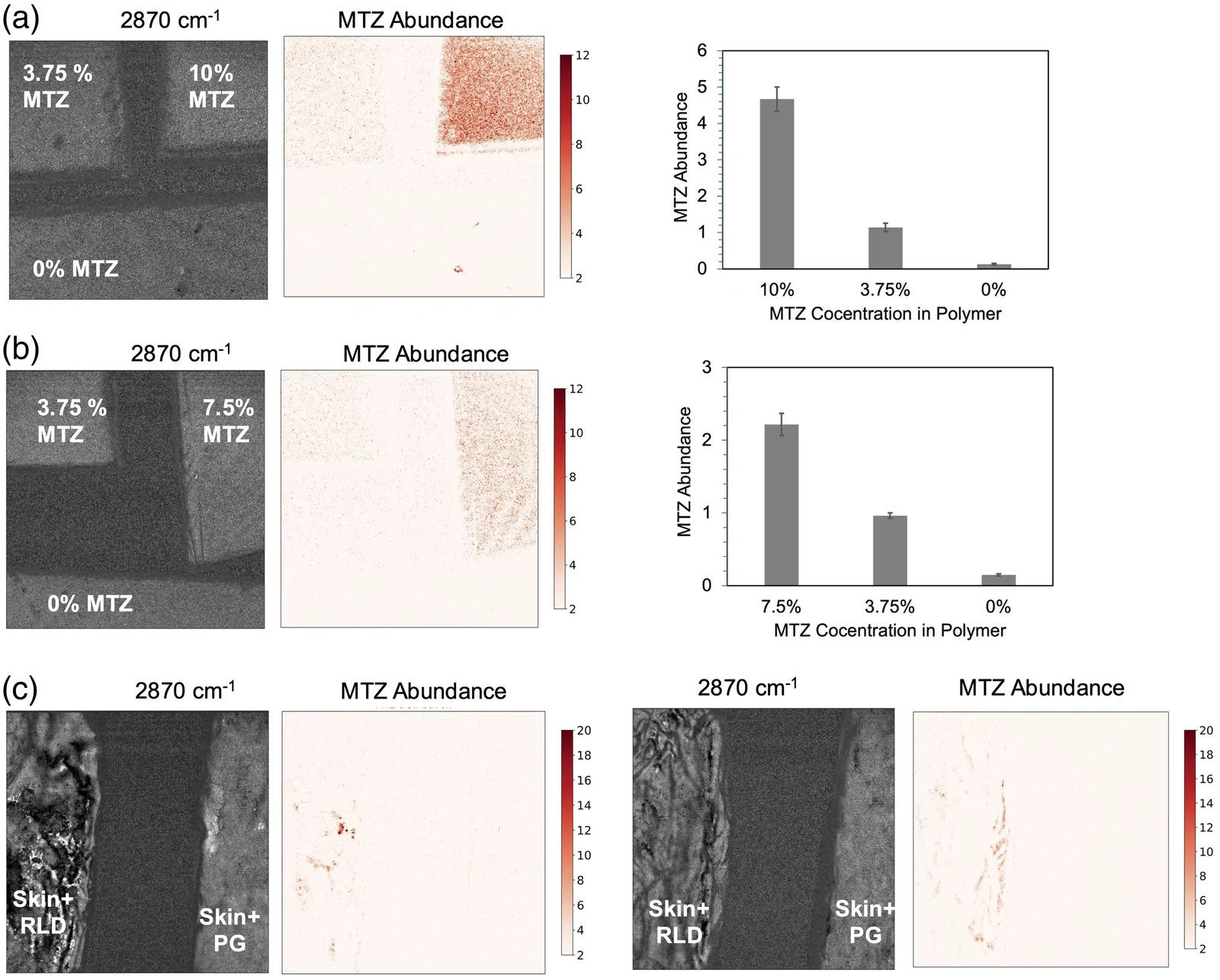
(a), (b) Visualization of metronidazole distribution in the polymer film standard embedded with different concentrations of metronidazole (0 to 10% w/w). The column plots used the intensity from the resolved abundance map. Error bars represent the standard deviation of values from three different rectangular areas. (c) Visualization of metronidazole distribution in skin samples: one piece of thin skin applied with 0.75% Metrogel^®^ drug product on the left, and the other piece of skin applied with propylene glycol on the right. The two pairs of images were from two separate experiments. In each pair of images, the left image shows the SRS image collected at $2870\;{\mathrm{cm}}^{-1}$, and the right image shows the resolved metronidazole abundance map using the collected S4RS image volume (17 wavenumbers). MTZ, metronidazole; RLD, reference listed drug; PG, propylene glycol.

Polymer films with different concentrations of metronidazole were used to evaluate the MCR-ALS analysis procedure for S4RS data processing. As shown in Figs. 3(a) and 3(b), polymer films with different concentrations of metronidazole were positioned in one field of view. The image at $2870\;{\mathrm{cm}}^{-1}$ shows the signal of the ${\mathrm{CH}}_{2}$ stretching vibration, an abundant vibrational band in PLA. The collected S4RS image volume for this field of view, which contained all 17 selected wavenumbers, was input to the MCR-ALS algorithm. The algorithm’s output was plotted as an abundance map of metronidazole components, which indicated the distribution of metronidazole within the image. The results demonstrate that metronidazole was correctly identified. Specifically, in regions where polymer films containing metronidazole were located, metronidazole was identified in the abundance map. Moreover, the high-concentration area had a higher abundance value than the low-concentration area. The values of different concentrations of polymer films were plotted and showed a clear change of abundance value due to changes in metronidazole within the films.

After confirming the performance of the analysis method using the controlled polymer film samples, the method was tested using thin skin applied with drug products. This sample contained all components present (the API and the interfering components) in the sample in actual experiments for BA/BE studies. In this test, a piece of thin skin was applied with 0.75% Metrogel^®^ drug product on the left, and another piece of skin was applied with propylene glycol on the right. The two samples were positioned close to each other so that they could be imaged in the same field of view. Propylene glycol is an excipient present in all tested formulations (RLD, generic product, and lab-made formulation) in this study. The experiment results are shown in Fig. 3(c). The image at $2870\;{\mathrm{cm}}^{-1}$ shows the skin microstructure based on the lipid (${\mathrm{CH}}_{2}$ stretch-rich) distribution. The abundance map of metronidazole components showed the presence of metronidazole on the left side of the image, corresponding to the skin area applied with the 0.75% Metrogel^®^. The observed heterogeneous distribution of metronidazole may have been caused by multiple factors, including the nonuniform gel distribution, creases in a nonperfectly flat skin sample resulting in out-of-focus areas, and precipitation of metronidazole after solvent evaporation. By contrast, on the right side of the image, where the skin was applied with an excipient, no metronidazole was identified. These results confirm that MCR-ALS combined with S4RS can identify the API in a complex sample matrix that contains all components present in actual experiments for BA/BE studies.

**Fig. 4:**
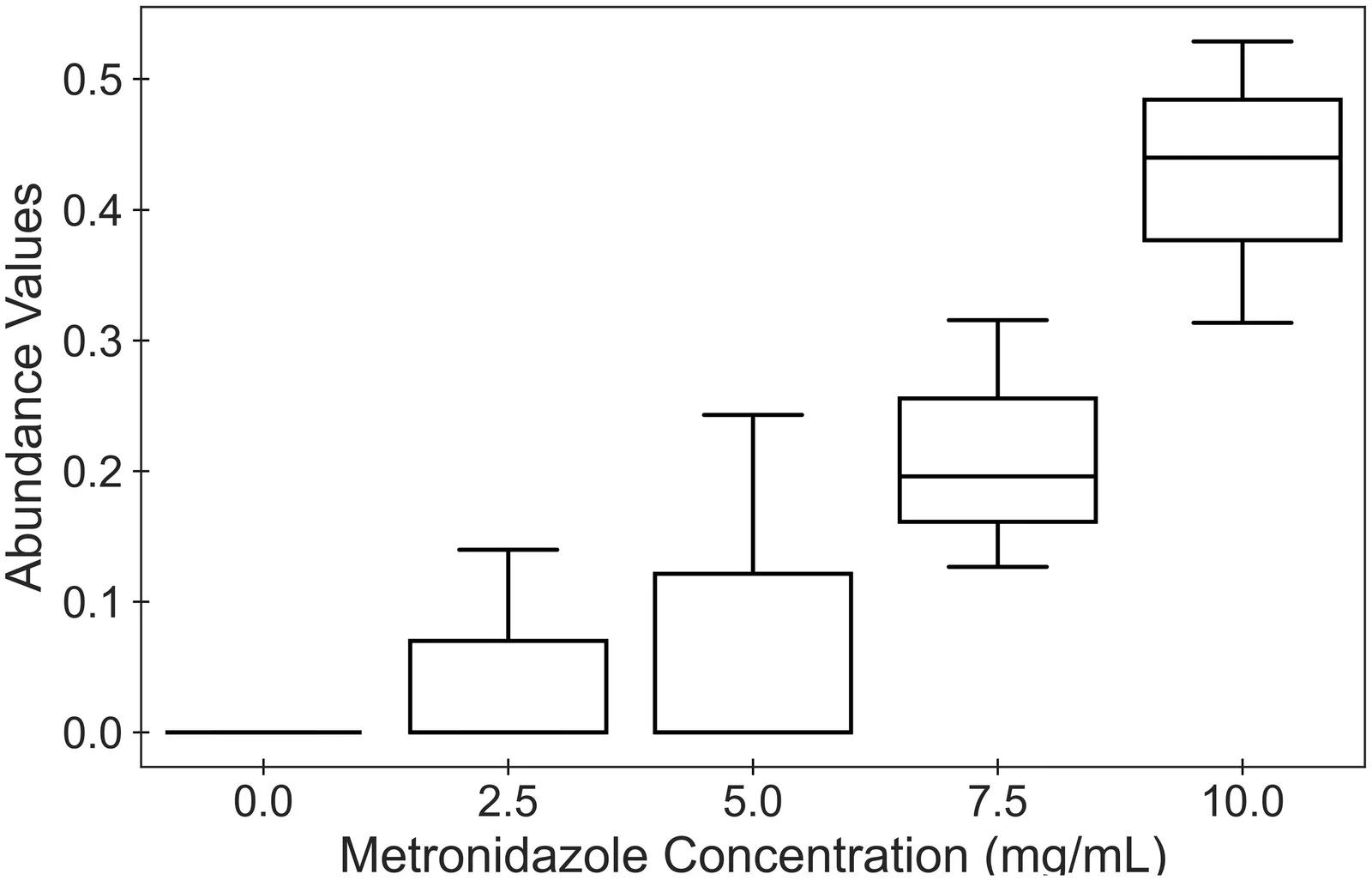
Box plot showing the change in the resolved metronidazole abundance values when varying the concentration of metronidazole in a solution (solvent is 90:10 v/v water/propylene glycol). Each concentration was acquired by three different measurements at different regions of a sample.

In another experiment, standard samples with different concentrations of API were tested to evaluate the performance of using the MCR-ALS combined with the S4RS method in quantifying the amount of dissolved API. As shown in Fig. 4, intensity values at the resolved metronidazole abundance increased with the concentration of metronidazole in the solvent. For higher concentration (7.5 and 10  mg/mL), the MCR-ALS analysis showed a good capability of identifying metronidazole and reflecting its concentration by abundance value. For solvent without any metronidazole, the output abundance is 0. However, wider variance in abundance values was observed for low-concentration metronidazole (2.5 and 5  mg/mL). This is due to some samples being output as zero abundance, and others being output with a nonzero value. These results may indicate that MCR-ALS combined with the S4RS method are more susceptible to noise when attempting to resolve a component at a lower concentration.

In summary, three different types of standard samples were used to assess the performance of the MCR-ALS algorithm in resolving the metronidazole signal from the S4RS image volume. These standard samples had known or stable compositions, which were helpful in evaluating the resolved results against the expected results. All observed results confirmed that the MCR-ALS method successfully resolved the metronidazole signal from a complex mixture, and the resolved signal intensity was correlated to the concentration of metronidazole present in the sample.

### 3.3 Multivariate Analysis on Long Time-Repeated Measurement

When monitoring API permeation in skin over long periods, it is important to ensure that the collected signal and its change are specifically related to API permeation. In a previous study using a single primary peak for tracking the API,^19^ it was observed that the intensity of SRS images varied during long time-repeated imaging due to fluctuations in the optical system or electronic noise in the modulating signal. An additional ROI with skin positioned with a standard polymer film was introduced to help overcome this problem. The additional ROI would experience the same fluctuation as the ROIs containing skin applied with the drug products because they were imaged in the same imaging cycles. This strategy has shown good performance in reducing signal fluctuations by normalizing the single peak intensity from the ROI with the drug products to that from the ROI with the polymer film.^19^

In this work, using a S4RS spectrum and multivariate analysis enabled us to overcome this challenge differently from the study using a single peak. Using multivariate analysis to resolve the API’s signal is advantageous, as it inherently accounts for signal fluctuations. In theory, MCR-ALS can account for components that are beyond the input reference component. Because of this, the latent fluctuating background components can be separated from the metronidazole signal. In this part, experiments were used to validate the capability of the MCR-ALS method combined with S4RS to reduce signal fluctuations during repeated measurements.

**Fig. 5:**
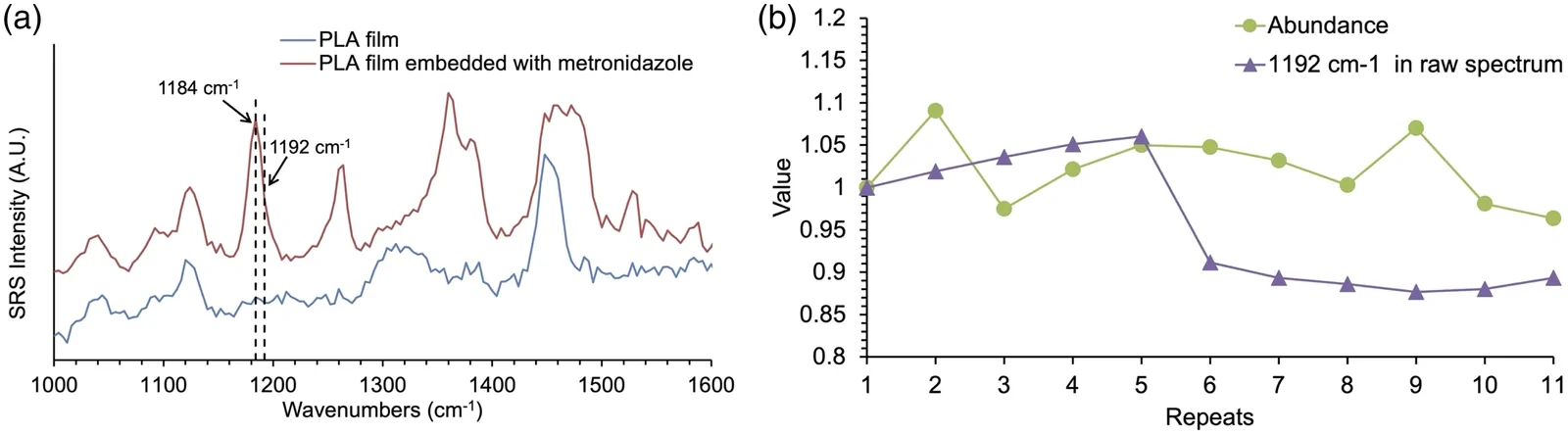
(a) Full SRS spectra of a PLA standard film embedded with 7.5% w/w metronidazole and a “blank” PLA film without metronidazole. (b) Intensity-time profile of a polymer standard film embedded with 7.5% w/w metronidazole. The same ROI of the same sample was measured using S4RS imaging repeatedly. The raw intensity at $1192\;{\mathrm{cm}}^{-1}$ of the signal is compared with the resolved metronidazole abundance value obtained from MCR-ALS and the 17-wavenumber S4RS spectrum.

To assess this aspect, an experiment using a polymer standard film embedded with metronidazole was imaged to acquire the 17-wavenumber S4RS spectrum. The same sample was imaged repeatedly to collect 11 sets of the 17-wavenumber S4RS spectrum. Because of the stability and inert feature of the standard polymer film, the composition of the film was constant throughout imaging. In addition, because only the polymer film was present without any skin or gel formulation, any signal variance was expected to be caused by imaging system variance or algorithm noise in the multivariate analysis, but not by chemical composition change. As shown in Fig. 5(a), metronidazole had a strong peak near $1184\;{\mathrm{cm}}^{-1}$. The peak position shifted compared to the peak at $1192^{-1}$ for metronidazole dissolved in a solvent. This difference in peak position was also observed in another study using confocal Raman spectroscopy.^42^ Although it was not the peak, the $1192\;{\mathrm{cm}}^{-1}$ was on the peak shoulder; therefore, the SRS intensity at $1192\;{\mathrm{cm}}^{-1}$ was still high and was more prominent compared to the blank PLA film. Based on this, the amount of metronidazole could be correlated with the intensity at $1192\;{\mathrm{cm}}^{-1}$. As shown in Fig. 5(b), $1192\;{\mathrm{cm}}^{-1}$ intensity in the raw S4RS spectra fluctuated (generally decreased) during the repeated measurements, which was expected due to imaging system variance, similar to what was observed in the previous study. By contrast, the resolved metronidazole abundance values using MCR-ALS showed a generally flat trend during repeated imaging. The abundance of metronidazole was expected to be constant throughout imaging; therefore, a flat line with a value of 1 is ideal. Although fluctuation (deviating most at point 2) was observed in the experimental data, the variances were between 0.97 and 1.09. This variance was much smaller than that observed in the single wavenumber intensity (0.88 to 1.06 for $1192\;{\mathrm{cm}}^{-1}$). The relatively small variance with MCR-ALS analysis indicates the feasibility of using resolved metronidazole abundance in a continuous study. Compared with using individual peak intensities in the spectrum, using the abundance value as the readout does not require normalization to an additional ROI with the polymer film.

### 3.4 Permeation Profiles of Drug Products in Ex Vivo Skin

**Fig. 6:**
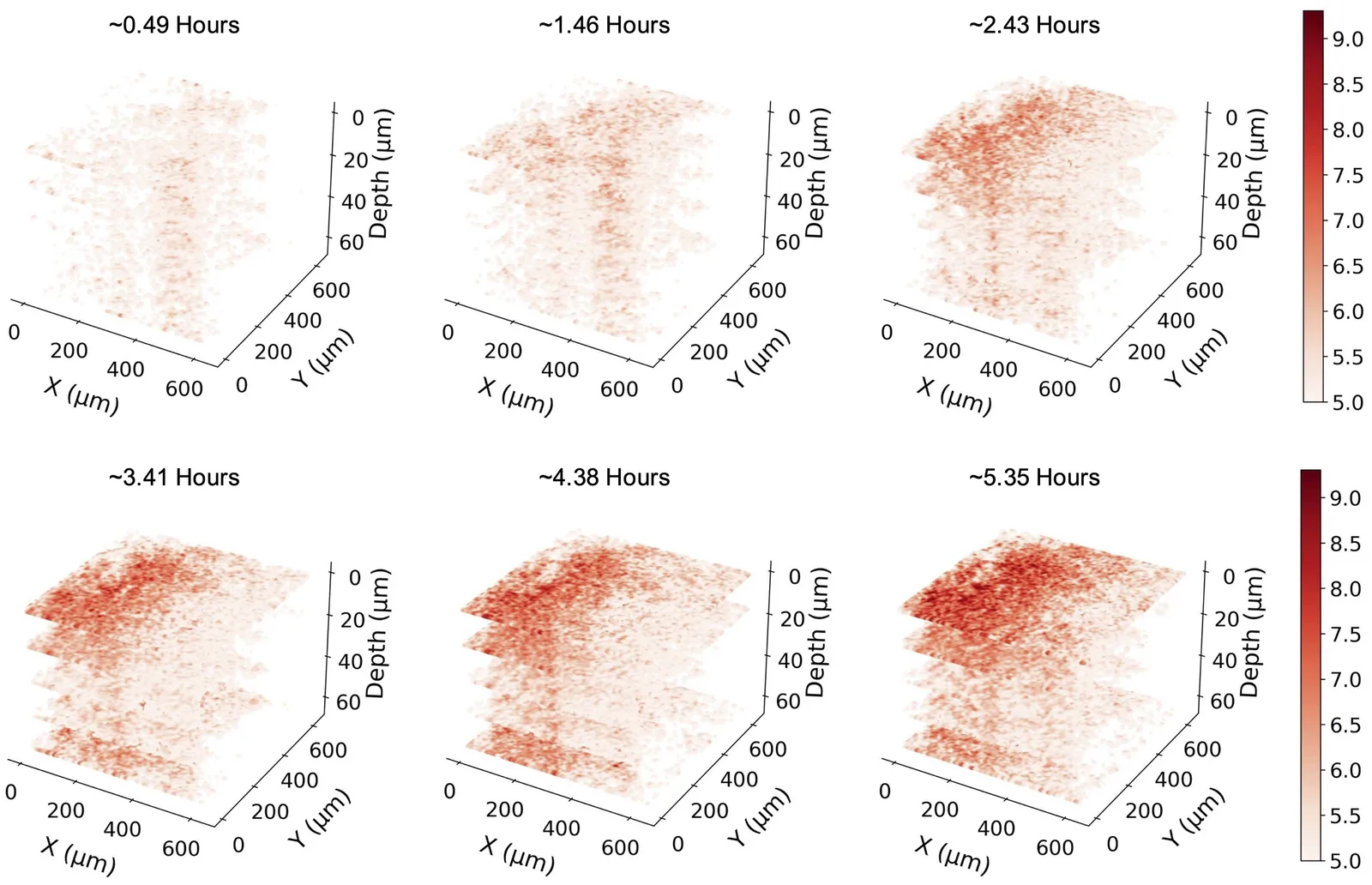
Monitoring of high-abundance areas at different depths (0 to 64  μm) of a skin sample during permeation of metronidazole. The skin sample shown was from a 52 year-old male donor. The laboratory-made formulation containing 0.75% metronidazole (solvent was 90:10 v/v water/propylene glycol) was applied to the skin. The raw abundance map was first preprocessed with a Gaussian filter to improve visualization. A threshold of 5 was used. Only pixels with abundance above 5 are shown.

The developed method enables visualization of the spatial distribution of an API. S4RS images collected at different depths of skin applied with drug products were processed by the MCR-ALS algorithm to generate a metronidazole abundance 3D map. As S4RS imaging is a nondestructive method, it enables repeated collection of the same area at different time points during drug permeation. Figure 6 shows the dynamic changes in the distribution of metronidazole across different depths over a five hour period.

**Fig. 7:**
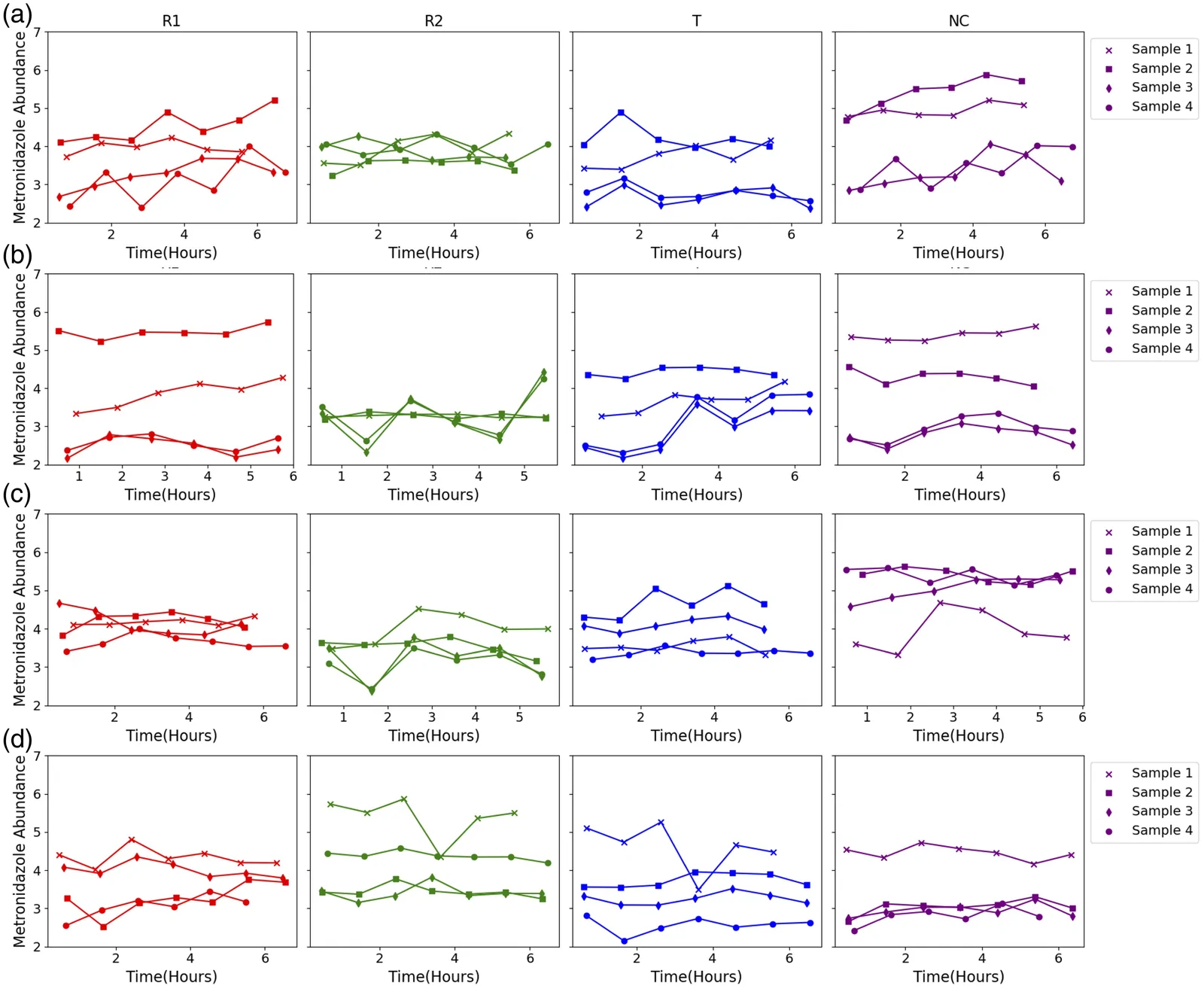
Metronidazole abundance value-time profiles collected from skin samples of different skin donors: (a) 52 year-old male, (b) 64 year-old female, (c) 41 year-old female, and (d) 38 year-old female. Plots in the four columns refer to the four treatment groups: R1 and R2 applied with 0.75% metronidazole gel RLD (Metrogel^®^), T applied with generic 0.75% metronidazole gel (Cosette Pharmaceuticals, Inc), and NC applied with laboratory-made formulation containing 0.75% metronidazole (solvent was 90:10 v/v water/propylene glycol). Each data point represents a value after averaging the values from three depths (0 to 32  μm) and three ROIs.

In addition, at each time point, the resolved metronidazole abundance in one ROI and at multiple depths can be averaged. The averaged intensity can be plotted against the drug application time, forming an intensity-time profile. Intensity profiles are useful in PK analysis because they contain rich information about the extent and rate of drug permeation through the skin. In this work, samples from four different skin donors were used: 52 year-old male, 64 year-old female, 41 year-old female, and 38 year-old female. The obtained intensity-time profiles are shown in Fig. 7. Each skin sample with one treatment was plotted as one profile, and the value in this profile was the average of values from three ROIs and two depth slices. Results for the four treatment groups were plotted individually. Two separate groups of data, labeled as R1 and R2, were measured using skin applied with the 0.75% metronidazole gel RLD (Metrogel^®^). One group of data, labeled as T, was measured using skin applied with the generic 0.75% metronidazole gel (Cosette Pharmaceuticals, Inc). The approved generic product was expected to have a similar extent and rate of metronidazole delivery in skin. The negative control (NC) group of data was measured using skin applied with the lab-made formulation containing 0.75% metronidazole (solvent was 90:10 v/v water/propylene glycol). Because the excipients comprising the lab-made formulation were different compared with those of the commercial products, it was expected to have a different rate and extent of metronidazole delivery in skin than the RLD.

For the 52 year-old male donor [Fig. 7(a)], the intensity-time profiles of group R1, R2, and T generally showed a small change over time with a slight increase in metronidazole abundance; the NC group, which was treated with the lab-made formulation, showed a higher bioavailability of metronidazole than the other treatment groups. The metronidazole abundance at the upper skin layers (0, 16, and 32  μm) and the deeper skin layers (48, 64  μm) were also compared. The difference in abundance between the two parts was plotted in Fig. S3A in Supplementary Material. It was observed that, in general, metronidazole in the deeper skin layers increased over time for all treatment groups. The difference values changed from a negative value at the first time point, indicating that the upper layers contained more API, to a positive value, indicating that the API permeated the deeper layers. This result confirmed the good permeation of these three tested formulations in skin samples from the 52 year-old male donor. The trend in the results of the 41 year-old female donor [Figs. 7(c) and S3C in Supplementary Material) was similar to that of the 52 year-old male, but with changes of smaller magnitude.

Profiles from the 64 year-old female donor [Fig. 7(b)] showed less difference in the abundance values across different treatment groups. Also, as shown in Fig. S3B in Supplementary Material, the difference between the upper skin layers and the deeper skin layers remained small over the 6-h time period. This indicated that the API had lower permeation into deeper layers, an anticipated intersubject variability.

In two profiles from the 38-year-old female donor [Fig. 7(d)], one being in the R2 and the other in the T group, clear troughs in profiles were observed. This type of crest and trough was also observed in Fig. 5, which was mainly caused by the noise of the S4RS combined with the MCR-ALS analysis method, but not by the decrease in metronidazole abundance. It is worth mentioning that the consecutive crests and troughs had opposite effects on the area under the curve values and thus were expected to cause limited change in the total area under the curve relative to the profile without them. Considering this, caution needs to be taken if using the developed method to reveal the absolute permeation profile. In comparative studies, including the BE study, the impact should be minimal and can be compensated for by averaging this type of noise through experimental replicates. Figure S3D in Supplementary Material shows the difference between the upper skin layers and the deeper skin layers for the 38 year-old female donor, where some samples showed high permeation of the API from upper layers to deeper layers over time. Overall in Fig. 7, noticeable variances were observed across samples from the same donor applied with the same drug products and also across profiles from different donors. This variance is not unexpected considering the difference between skin donor subjects and the heterogeneity of skin samples from the same donor, as well as the microscale variability in hand-trimmed skin. Notably, the current study used four replicates for each donor, which is a relatively small sample size. More replicates may be helpful to comprehensively reveal the permeation profiles for each treatment group.

The time interval between two points in Fig. 7 is ∼1  h, a period determined by the experimental design. In the utilized design, one image volume of an ROI was collected at a resolution of 512×512  pixels, with a 10  μs pixel integration time, and a five-slice Z-stack (0 to 64  μm, with a 16  μm step size). A total of eight ROIs were imaged, and each ROI was imaged with 19 wavenumbers through several loops over the time course. In this setup, each one five-slice Z-stack took around 8 min to complete the scan of 19 wavenumbers scan, including the delay associated with movement between Z-stack positions and the microscope’s Z-drift correction. If the number of wavenumbers increased, the acquisition time for a single point in the time profile would also increase. For example, if 30 wavenumbers were selected, it was estimated that the acquisition time for one data point would increase to 1.5 h. The long acquisition time would sacrifice the time resolution in capturing changes in bioavailability during API permeation. Therefore, fewer selected wavenumbers were favored for a shorter acquisition time and a smaller time interval. Considering these aspects, the number of selected wavenumbers was set to be less than 30 in the workflow shown in Fig. S1 in Supplementary Material. This value was chosen based on the estimation of imaging time based on the selected integration time, the pixel resolution, the number of Z-slices, and the number of ROIs used in the experiment. If any of these experimental parameter amounts are reduced, the number of wavenumbers can be increased to exceed 30 while preserving the total acquisition time and maintaining good time resolution.

### 3.5 Bioequivalence Analysis of Drug Products

BE analysis was implemented using the permeation data collected above. Four treatment groups were evaluated in pairs. Using the obtained abundance value-time profiles, the AUC and $C_{\max}$ values could be calculated. The AUC and $C_{\max}$ values were then used to calculate the confidence interval (CI) when comparing two treatment groups. The calculated CIs of all treatment groups are listed in Table 1.

**Table 1:** BE analysis of the log-transformed $C_{\max}$ and AUC between drug products in the upper skin layers (0 to 32  μm). The $C_{\max}$ and AUC were calculated using the resolved metronidazole abundance value-time profiles. The output CIs were converted back to non-log-transformed values and listed as (Low limit, high limit).

Product 1	Product 2	90% Confidence interval for the mean difference of the $C_{\max}$ values (Product 2 – Product 1)	90% Confidence interval for the mean difference of the AUC values (Product 2 – Product 1)
RLD (R1)	RLD (R2)	(0.88, 1.11)	(0.81, 1.11)
RLD (R1)	Generic	(0.86, 1.10)	(0.85, 1.09)
RLD (R1)	Lab ($H_{2}O/\mathrm{PG}$)	(0.90, 1.27)	(0.93, 1.25)
RLD (R2)	Generic	(0.85, 1.15)	(0.83, 1.24)
RLD (R2)	Lab ($H_{2}O/\mathrm{PG}$)	(0.82, 1.43)	(0.84, 1.53)

When comparing the R1 with the R2 group, all CIs were within the BE limit (0.8 to 1.25), indicating the bioequivalence of these two groups. The R1 and R2 groups were measured using skin applied with the same RLD product. Comparison of R1 and R2 helps to evaluate the inter-experiment variance of the developed S4RS method combined with the multivariate analysis method. Bioequivalence between R1 and R2 using only four donors indicates the relatively low inter-experiment variance when using the developed method for BE analysis.

When comparing the T group with the R1 or R2 group, all CIs were within the BE limit, indicating their bioequivalence. The tested generic 0.75% metronidazole gel (Cosette Pharmaceuticals, Inc) is a commercially available, approved generic drug that has been shown to be bioequivalent to the RLD. The results obtained here matched the expected bioequivalence between the generic product group and the RLD product group.

When comparing the NC group with the R1 or R2 group, the CIs were outside of the BE limit, indicating that the lab-made formulation was not bioequivalent to the RLD. The lab-made formulation consisted of 90:10 v/v water/propylene glycol as a solvent, which differ from the RLD. The different compositions led to different permeation profiles and, consequently, to non-BE between the lab-made formulation and the RLD. The successful determination of both BE and non-BE relations confirmed the capability of the developed imaging method to study drug products and its potential for BE assessment.

## 4 Conclusion

This work developed a method based on S4RS imaging and multivariate analysis to resolve the signal of an API in cutaneous permeation studies. Metronidazole was used as a model molecule to demonstrate and validate the developed method. S4RS acquires a sparse set of representative wavenumbers for the molecule, which helps to preserve key molecular information while enabling a short acquisition time when using sequential-tuning lasers. The feature selection method, elastic net, successfully determined 17 wavenumbers that were representative of metronidazole and which were sufficient to distinguish metronidazole from skin and other molecules present in the drug products. The MCR-ALS analysis, combined with S4RS imaging using 17 wavenumbers, was validated using standard samples. Three types of standard samples with known and stable compositions were used to evaluate the performance of the developed multivariate analysis-assisted S4RS imaging. The results demonstrated that the imaging method can successfully resolve the metronidazole signal from that of mixture samples, and the output value was correlated to the concentration of metronidazole present in the sample. In addition, the repeated measurement experiment confirmed the relatively good signal stability of the developed method in long time period repeated measurement. Signal stability in this method is important, as it ensures that the observed trend in the intensity-time profile is specifically related to API permeation. Using MCR-ALS analysis, the resolved metronidazole abundance exhibited much smaller signal fluctuations than when using single-peak intensity. The multivariate analysis method accounted for the high signal variance arising from electronic noise or fluctuations in the optical system, which presents a main challenge in studies using a single primary peak. As a result, an additional ROI for signal normalization to compensate for these fluctuations was not required. Despite the demonstrated capability of the developed method, there are limitations to this approach. The current method performs better at identifying areas where metronidazole concentration is high, but can encounter limited performance when the concentration becomes low (≤5  mg/mL, around 0.5% w/w). Future work will focused on further improving the method for low-concentration signal resolution.

In the last part of this study, BE analysis of four treatment groups was carried out using the developed method. The R1 and R2 groups, which were treated with the same RLD product, were determined to be BE. The BE of the R1 and R2 groups confirmed low inter-experiment variance when using the developed method. The establishment of BE between the RLD and the approved generic product and the non-BE between the RLD and the lab-made formulation demonstrated the potential of the developed imaging method for prospective BE studies. In this work, the wavenumber selections and the results were demonstrated for metronidazole, but the workflow and methods are generalizable and can be applied to other drug products containing different APIs. Moreover, because the method uses spectral information rather than a single primary peak corresponding to a unique chemical band in the API, its scope extends to APIs that lack a single, unique, and strong peak. Therefore, this work is an important step toward enabling the use of the stimulated Raman-based technique in the BE study of a broader range of drug products.

## Supplementary Material

10.1117/1.BIOS.3.4.043103.s01

## Data Availability

The data related to the manuscript are publicly available through the Harvard Dataverse repository at https://doi.org/10.7910/DVN/WUWAUC.

## Author Credit Statement

Dandan Tu: Conceptualization, Methodology, Formal analysis, Visualization, Writing—Original draft preparation. Jackson Riseman: Methodology, Investigation, Data acquisition, Writing—Reviewing and Editing. Yuxiao Wei: Methodology, Writing—Reviewing and Editing. Priyanka Ghosh: Conceptualization, Writing—Reviewing and Editing. Sam G. Raney: Conceptualization, Writing—Reviewing and Editing. Markham C. Luke: Conceptualization, Writing—Reviewing and Editing. Conor L. Evans: Conceptualization, Resources, Supervision, Project administration, Funding acquisition, Writing—Reviewing and Editing
