# Supplementary material for: Label-free quantification of topical drug permeation in skin using sparse spectral sampling stimulated Raman scattering imaging
Source: Biophotonics Discov. 2026 Aug 31;3(4):043103. doi: 10.1117/1.BIOS.3.4.043103 (PMC13557456; doi:10.1117/1.BIOS.3.4.043103)
Supplement: Supplementary file 1 [file BIOS_003_043103_SD001.pdf]

Supplementary Information: Label-free imaging of topical drug permeation in skin using sparse spectral sampling stimulated Raman scattering (S4RS) microscopy

Dandan Tu[1], Jackson Riseman[1], Yuxiao Wei[1], Priyanka Ghosh[2], Sam G. Raney[2], Markham C. Luke[2], and Conor L. Evans*[1]

[1]Wellman Center for Photomedicine, Massachusetts General Hospital, Harvard Medical School, Boston, 02114, USA
[2]Office of Research and Standards, Office of Generic Drugs, Center for Drug Evaluation and Research, U.S. Food and Drug Administration, Silver Spring, MD 20993, USA
*Corresponding author, Email: evans.conor@mgh.harvard.edu

1 Flow chart for S4RS assisted with multivariate analysis

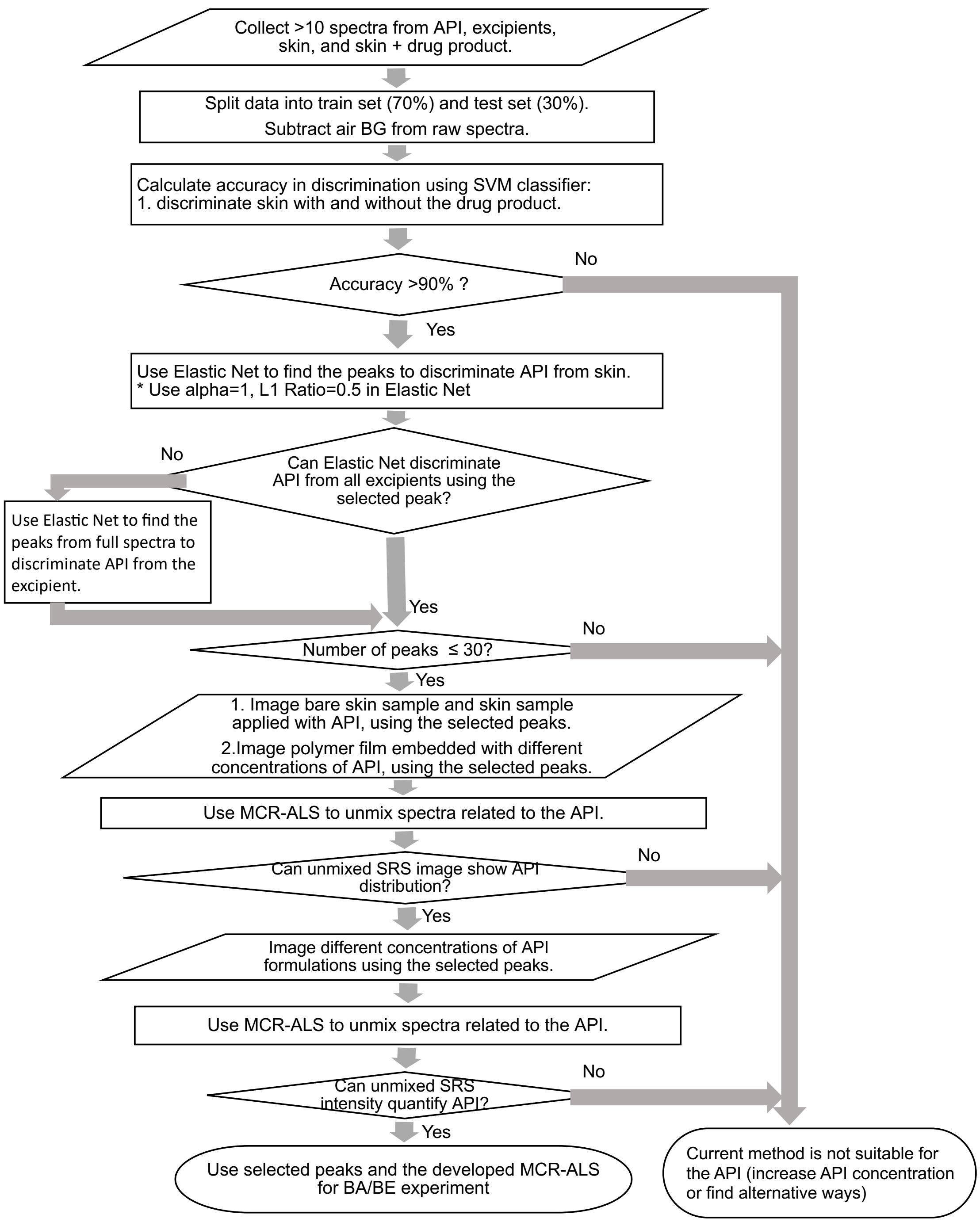

Figure S1: Flow chart for studying active pharmaceutical ingredient (API) in a drug product based on multivariate analysis and sparse spectral sampling stimulated Raman scattering (S4RS) imaging. BG: background. SVM: support vector machine. MCR-ALS: multivariate curve resolution - alternating least squares. BA: bioavailability. BE: bioequivalence.

2 Elastic Net results

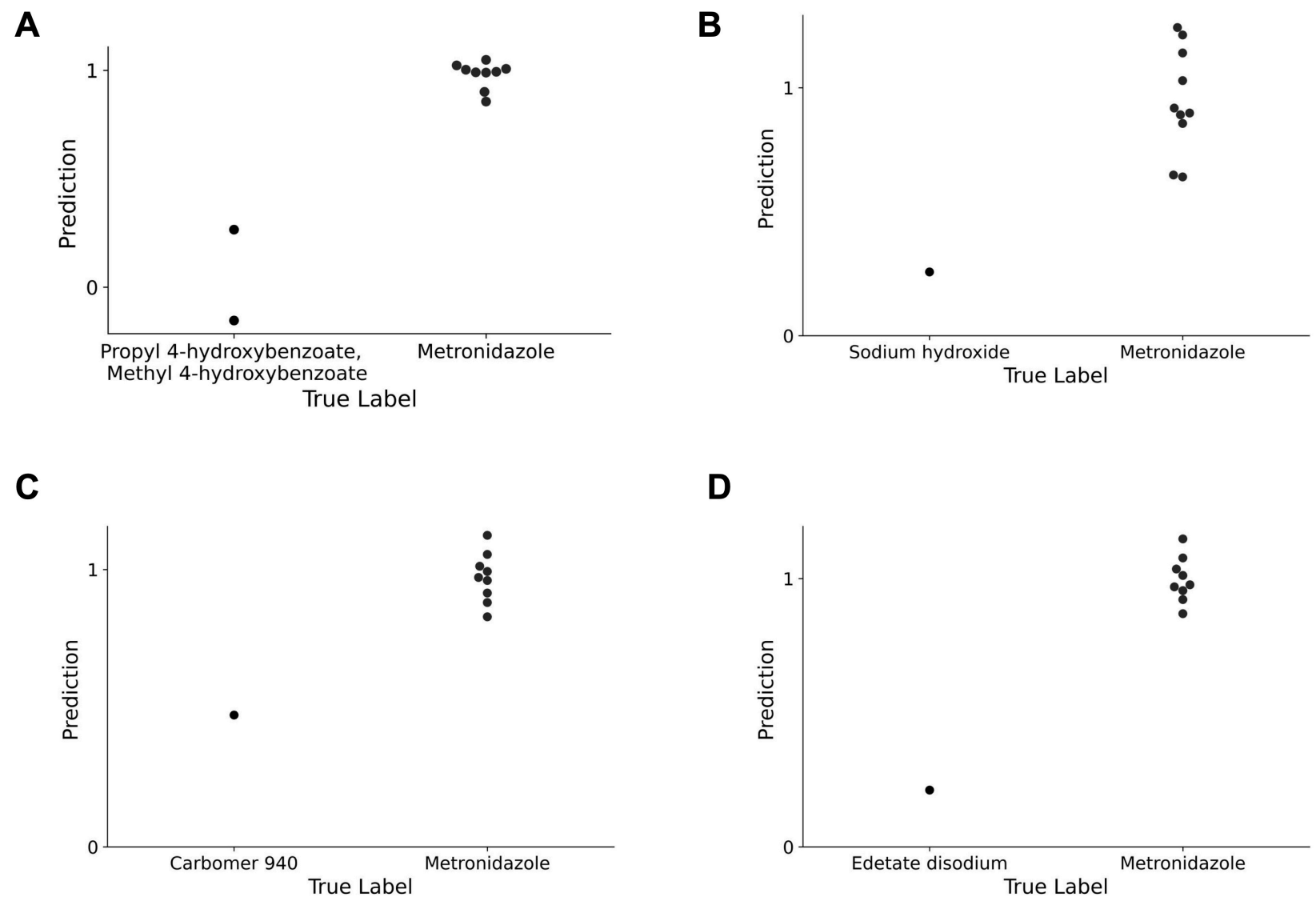

Figure S2: Elastic Net results using the 17 selected peaks for differentiating metronidazole from propyl 4-hydroxybenzoate, methyl 4-hydroxybenzoate, sodium hydroxide, carbomer 940, and edetate disodium. The predicted value of 0 indicates no presence of metronidazole, and a value of 1 indicates the presence of metronidazole.

3 Polymer film optimization

Table S1: Solubility of metronidazole in different solvents.

**Solvent**	Is 15 mg/mL MTZ dissolved immediately?
Dichloromethane	No
Methanol	Yes
Ethyl Acetate	No
Tetrahydrofuran	Yes
Ethanol	No
Acetone	No
Acetonitrile	No
Water	≤ 5.9 mg/mL
Water + Methanol	≤ 9.2 mg/mL
Dimethyl Sulfoxide	Yes

Table S2: Trials of polymer film production using different solvents and polymers. PPMA: poly(n-propyl methacrylate). PMMA: poly(methyl methacrylate). PLGA 75:25: poly(D,L-lactide-*co*-glycolide), ester-terminated, 75:25 copolymer ratio. PVA: poly(vinyl alcohol). P(D,L)LA: poly(D,L-lactide) approximately 1:1 ester:acid-terminated. MTZ: metronidazole. Sci. Poly. : SciPoly. Sig. : Millipore-Sigma.

	**Solvent**	**PPMA (150 kDa, Sci. Poly. #828)**	**PMMA (75 kDa, Sci. Poly. #037B)**	**PLGA (66-107 kDa, 75:25 lactide:glycolide, Sig. #P1941)**	**PVA (9-10 kDa, 80% hydrolyzed, Sig. #360627)**	**P(D,L)LA, (75-120 kDa, Sig. #P1691)**
**Polymer Only (without MTZ)**	Dimethyl Sulfoxide	no film; polymer insoluble	no film; polymer insoluble	transparent, fragile film	transparent film	transparent film
	Tetrahydrofuran	transparent film	transparent film	no film; polymer disintegrated	no film; polymer insoluble	fragile, translucent film
	Methanol	no film	no film	no film; polymer insoluble	transparent film<br><br>*Solvent is 43% methanol and 57% deionized water	no film; polymer insoluble
**Polymer embedded with MTZ**	Dimethyl Sulfoxide				transparent film; polymer dissolves with on-tissue experiments	transparent film
	Tetrahydrofuran	minor drug crystallization in film	minor drug crystallization in film			
	Methanol				transparent film; polymer dissolves with on-tissue experiments<br><br>*Solvent is 43% methanol and 57% deionized water	

A new method was developed to manufacture a polymer standard film suitable for metronidazole, a hydrophilic API with limited solubility in pure water. A variety of solvents were tested to determine which would dissolve high concentrations of metronidazole, with the ideal solvent being volatile for fast and room-temperature film drying. Upon initial tests, methanol was the only volatile solvent that easily dissolved metronidazole, while tetrahydrofuran (THF) dissolved it after sonication. Dimethyl sulfoxide (DMSO) dissolved the API easily, but it is not volatile.

Next, these three solvents with metronidazole solvation ability were tested to assess their ability to form a transparent film and to solvate polymer bases. In subsequent experiments, the combinations of polymer base and solvent that could form a transparent film were tested to assess their ability to form a stable, transparent metronidazole-embedded film. Observational results of these tests are summarized in Table S2.

As shown in Table S2, none of the polymers tested were effective in forming a stable metronidazole-embedded film using pure methanol. Among the listed polymers, because poly(vinyl alcohol) is a highly water-soluble polymer, a solvent mixture of both water and methanol was also tested with poly(vinyl alcohol). It was observed that metronidazole in pure water dissolved to a maximum of approximately 5.9 mg/mL, and dissolved more readily in a separate test in which methanol was added to a 10 mg/mL API solution in pure water up to 43% methanol volumetrically. The API solubility after co-dissolution in methanol was approximately 9.2 mg/mL. Using this water+methanol solvent, the produced poly(vinyl alcohol) films with embedded metronidazole were stable and transparent.

However, the film did not remain intact during a tissue-permeation experiment; skin moisture was sufficient to dissolve the film either partially or completely over the course of the imaging series.

As THF was another promising volatile solvent, we tested film production with various polymers. The most hydrophilic polymers, such as PVA and polymers of lactic acid and glycolic acid, dissolved poorly in THF, and the resulting films disintegrated upon drying. The hydrophobic polymers poly(n-propyl methacrylate) (PPMA) and poly(methyl methacrylate) (PMMA) produced high-quality films in THF at both 5% and 10% polymer concentration by solvent volume, without metronidazole. However, when metronidazole was included, it did not remain embedded after solvent evaporation and precipitated as API crystals.

Although DMSO is not a volatile solvent, in contrast to methanol and THF, it was the optimal solvent for forming a stable, intact, and transparent metronidazole-embedded film. An additional heating step was used to accelerate solvent evaporation during film formation. After dissolving a polymer in DMSO at 5% weight by volume, with or without metronidazole, the precursor solution was briefly sonicated, mixed by vortexing, and cast into wells of poly(dimethyl siloxane) molds on large glass slides. Rather than drying at room temperature when using methanol and THF as the solvent, the DMSO mixture was placed in a tabletop convection/hybridization oven set to 70 °C for two hours. As summarized in Table S2, poly(D,L-lactide-*co*-glycolide) (PLGA) produced fragile films, and poly(D,L-lactide) (PLA) produced robust, reproducible, transparent films with DMSO, both with and without embedded metronidazole. The highest achievable metronidazole concentration in the film before significant crystallization was 15 mg per mg of polymer.

4 Difference in permeation profiles between skin depths

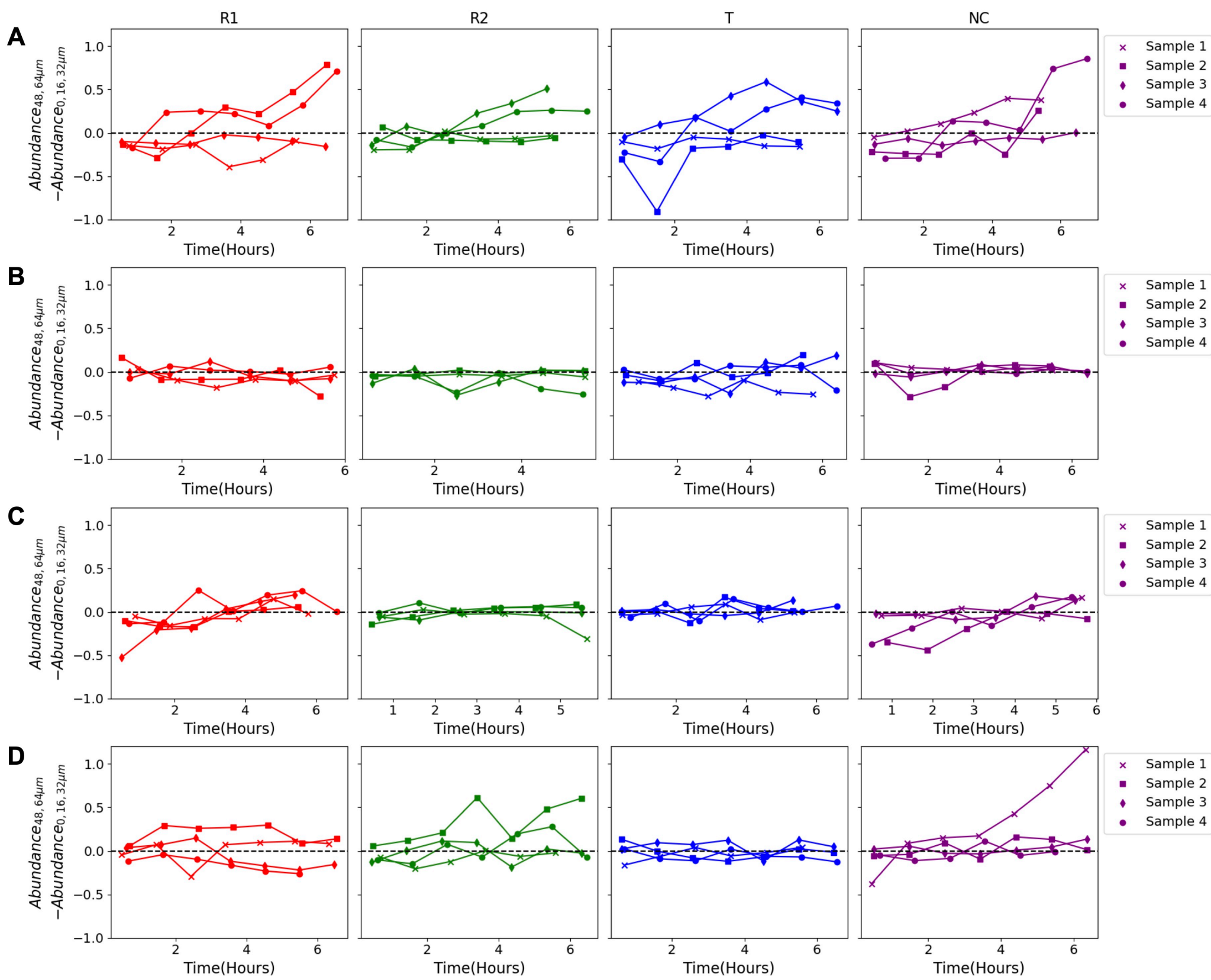

Figure S3: Difference between metronidazole abundance at deeper skin depths (48, 64 μm) and upper skin depths (0, 16, 32 μm). Results from different donors were plotted separately: (A) 52 year-old male, (B) 64 year-old female, (C) 41 year-old female, and (D) 38 year-old female. Plots in the four columns refer to the four treatment groups: R1 and R2 applied with 0.75% metronidazole gel RLD (Metrogel®), T applied with generic 0.75% metronidazole gel (Cosette Pharmaceuticals, Inc.), and NC applied with laboratory-made formulation containing 0.75% metronidazole (solvent was 90:10 v/v water/propylene glycol). Each data point represents a value after averaging the values from different depths and three ROIs.
